# Delaware’s New Medical Law

**Published:** 1895-09

**Authors:** 


DELEWARE has a new medical law. It provides for two boards of examiners, regular and homeopathic medicine being represented, and imposes a fine of $500 or not more than one year's imprisonment for persons convicted of practicing without a license. The law went into effect in July.

